# Hodgkin's Disease in Patients with HIV Infection

**DOI:** 10.1155/2011/402682

**Published:** 2010-09-23

**Authors:** Michele Spina, Antonino Carbone, Annunziata Gloghini, Diego Serraino, Massimiliano Berretta, Umberto Tirelli

**Affiliations:** ^1^Division of Medical Oncology, National Cancer Institute, via Franco Gallini 2, 33081 Aviano (PN), Italy; ^2^Division of Pathology, National Cancer Institute, via Franco Gallini 2, 33081 Aviano (PN), Italy; ^3^Department of Pathology, and Laboratory Medicine, Fondazione IRCCS, National Cancer Institute, via Giacomo Venezian 1, 20133 Milan, Italy; ^4^Unit of Epidemiology and Biostatistics, National Cancer Institute, via Franco Gallini 2, 33081 Aviano (PN), Italy

## Abstract

Hodgkin lymphoma (HL) represents one of the most common non-AIDS-defining cancers with an increasing incidence overtime. Clinically, patients present advanced stages of disease with extranodal involvement in the majority of cases. In the last years, significant improvements in the treatment of patients with HL and HIV infection have been achieved. In the lack of randomized trials, several phase II studies have showed that in the era of highly active antiretroviral therapy (HAART) the same regimens employed in HIV-negative patients with HL can be used in HIV setting with similar results. Moreover, in the last years the feasibility of high dose chemotherapy and peripheral stem cell rescue has allowed to save those patients who failed the upfront treatment. Finally, in the near future, a better integration of diagnostic tools (including PET scan), chemotherapy (including new drugs), radiotherapy, HAART, and supportive care will significantly improve the outcome of these patients.

## 1. Introduction

The availability of highly active antiretroviral therapy (HAART) has led to improvements in immune status among HIV-infected persons, reducing AIDS-related morbidity and prolonging survival. However, despite the impact of HAART on HIV-related mortality, malignancies remain an important cause of death in the current era [1, 2]. The use of HAART was also associated with reduced incidence of the two major AIDS-associated malignancies—Kaposi's sarcoma (KS) and high-grade non-Hodgkin lymphoma (NHL) [3]. However, among non-AIDS-defining cancers, an increased risk of Hodgkin lymphoma (HL), anal cancer, lung cancer, and hepatocarcinoma has been recently observed [4]. 

Although HL is included in the World Health Organization's categorisation of HIV-associated lymphomas [5, 6], the relation between HIV infection, AIDS, and HL is unclear.

HIV-associated HL (HIV-HL) displays several peculiarities when compared with HL of the general population. First, HIV-HL exhibits an unusually aggressive clinical behavior, which mandates the use of specific therapeutic strategies and is associated with a poor prognosis. Second, the pathologic spectrum of HIV-HL differs markedly from that of HL in the general population [7, 8]. In particular, the aggressive histological subtypes of classic HL (cHL), namely, mixed cellularity (MC) and lymphocyte depletion (LD), predominate among HIV-HL and the tumor tissue are characterized by an unusually large proportion of neoplastic cells, termed Reed-Sternberg (RS) cells [7]. Finally, despite the great improvement in chemotherapy and supportive care, optimal staging and treatment is still a matter of controversy.

## 2. Epidemiology

In HIV-negative population of the western countries, HL is one of the commonest malignancies diagnosed in young adults with 6 cases per 100.000 inhabitants under 45 years of age occur each year [9], even if an increase in incidence rate in the last decade has been observed [10]. The epidemiology of HL is characterized by a peculiar age distribution pattern—a bimodal incidence curve with a first peak around the age of 30 and the second peak around the age of 50 years—that has been taken as suggestive of an infectious etiology.

In immune suppressed patients, HL occurs more frequently than in the general population of the same age and gender. Given the relative high frequency of HL in the population groups at high risk for HIV infection, epidemiological studies conducted during the first years of the HIV epidemic in North America and in Europe had difficulties in including HL in the spectrum of HIV-associated cancers. However, with the spread of the epidemic and longer survival of infected people, the impact of HL could be better recognized. All studies [4, 11–22] strongly support the evidence that HIV-infected persons have, overall, a 10-fold higher risk of developing HL than HIV-negative persons. HL in HIV-infected individuals is more frequent in patients with moderate immune suppression and this is in sharp contrast with KS and diffuse large B-cell NHL that typically arise in a more pronounced immunosuppression setting [4]. Thus, the epidemiological pattern of HL in HAART era substantially differs from those observed for KS or NHL—two neoplasms which drastically decreased after the introduction of HAART—and pose several new questions with regard of the relationship between degree of immunodeficiency, persistent viral infections and cancer. Of some interest is the recent observation of Powles et al. who have investigated the occurrence of cancers in a prospective cohort of 11,112 HIV-positive individuals, with 71,687 patient-years of followup [23]. 

Standardized incidence ratios (SIRs) were calculated using general population incidence data. The incidence of HL in the HIV cohort was higher than in the general population (SIR 13.85; 95% CI, 9.64 to 19.26). There was a significant increase in the SIRs across the three study periods (1983 to 1995: 4.5; 1996 to 2001: 11.1, and 2002 to 2007: 32.4). Multivariate analysis demonstrated that HAART was associated with an increased risk of disease (SIR 2.67; 95% CI, 1.19 to 6.02). Further multivariate modeling by class of antiretroviral agents showed that of the three classes of antiretroviral therapy, only the Non-Nucleoside Reverse Transcriptase Inhibitors were associated with a significant increase in the incidence of HIV-HL (HR 2.20; 95% CI, 1.03 to 4.69). As the overall effect of HAART is to increase the CD4 count level, it paradoxically could increase HL incidence. A potential mechanism emphasizes the role of the RS cells producing several growth factors that increased the influx of CD4 cell and inflammatory cells, which, in turn, provide proliferation signals for the RS neoplastic cells. One can imagine that in the case of severe immune suppression, leading to an unfavorable milieu, the progression of the RS neoplastic cells can be compromised [24–26]. In addition, HIV-HL is EBV–associated in almost all cases, in contrast to what was observed in the general population, in which this association is only observed in 20% to 50% according to histological type and age at diagnosis [27]. Usurpation of physiologically relevant pathways by EBV-encoded latent membrane protein 1 (LMP1) may lead to the simultaneous or sequential activation of signaling pathways involved in the promotion of cell activation, growth, and survival, contributing thus to most of the features of HIV-HL. Whether this change affects its categorization as HL or whether it delays HL development is unknown. 

In summary, the use of HAART has improved the immunity of HIV-infected persons, diminishing the risks of developing other cancers or other opportunistic infections and paradoxically increasing the risk of HL. 

## 3. Pathological Features

In the pre HAART era, HIV-HL displayed different pathological features in comparison with those of HL in HIV-negative patients [7]. In fact, HIV-HL was characterized by the high incidence of unfavorable histological subtypes (i.e., MC and LD) [7, 8]. Among HIV-infected persons, MC was the most frequent HL subtype whereas nodular sclerosis (NS) was less frequent than in HIV-uninfected persons. For each HL subtype, incidence decreased with declining CD4 counts, but NS subtype decreased more precipitously than MC subtype, thereby increasing the proportion of MC subtype of HL seen in persons with HIV/AIDS. Thus, the greater proportion of MC and LD subtypes appeared specifically related to severe immune compromise in HIV, while in HAART era HIV-infected patients with modest immune compromise are more at risk for the development of the NS subtype [4]. CHL is a monoclonal lymphoid neoplasm, derived from B cells, composed of mononuclear Hodgkin cells and multinucleated Reed Stenberg (HRS) cells residing in a cellular microenvironment. HRS cells consistently express CD30 and CD40 and express CD15 in the majority of cases. CD20 is positive in a minority of neoplastic cells in 30–40% of cases, while the plasma cell-specific transcription factor Interferon Regulatory Factor 4 (IRF4) is consistently positive in HRS cells [5, 8]. 

On the other hand, HIV-HL may exhibit special features related to the cellular background (presence of fibrohistiocytoid stromal cell proliferation) and the high number of the neoplastic cell. Both these features may pose relevant difficulties in diagnosing and classifying the disease precisely. In these cases, a large cell lymphoma of the anaplastic cell type must be excluded on immunohistologic ground [7, 28]. 

A high frequency of EBV association has been shown in HL (80–100%) tissues from HIV-HL [29, 30]. The EBV genomes in such cases have been reported to be episomal and clonal, even when detected in multiple independent lesions. The elevated frequency of EBV association with HIV-HL indicates that EBV probably does represent a relevant factor involved in the pathogenesis of HIV-HL. An etiologic role of EBV in the pathogenesis of HIV-HL is further supported by data showing that LMP-1 is expressed in virtually all HIV-HL cases [5, 28–31]. On these bases, HL in HIV-infected persons appears to be an EBV-related lymphoma expressing LMP1. 

Finally, RS cells of classical HL-of HIV-negative patients represent transformed B-cells that originate from preapoptotic germinal center (GC) B-cells [32]. Most HIV-related HL cases express LMP1 and display the BCL6-/CD138+/MUM1 IRF4+ (for Multiple Myeloma-1 Interferon Regulatory Factor-4), phenotype, thus reflecting post-GC B cells [29, 32]. The possible contribution of LMP1 to the loss of BCL6 expression seems plausible given that LMP1 can downregulate many B-cell specific genes [33]. Loss of B-cell identity occurs during the normal differentiation of a GC B-cell into plasma cell or memory B-cell. 

## 4. Clinical Aspects and Treatment

Similar to that observed in HIV-NHL, one of the most peculiar features of HIV-HL is the widespread extent of the disease at presentation and the frequency of systemic “B” symptoms, including fever, night sweats, and/or weight loss >10% of the normal body weight. At the time of diagnosis, 70–96% of the patients have “B” symptoms and 74–92% have advanced stages of disease with frequent involvement of extranodal sites, with the most common being bone marrow (40–50%), and liver (15–40%) and spleen (around 20%) [8, 34–36]. HIV-HL tends to develop as an earlier manifestation of HIV infection with higher median CD4+ cell count. [8, 34–36]. The widespread use of HAART has resulted in substantial improvement in the survival of patients with HIV infection and lymphomas, due to the reduction of the incidence of opportunistic infections, the opportunity to allow more aggressive chemotherapy, and the less aggressive presentation of lymphoma in patients in HAART in comparison with those lymphomas diagnosed in patients who never received HAART [8, 34–37].

Within the Italian Cooperative Group on AIDS and Tumors (GICAT), we have collected data on 290 patients with HIV-HL. Two hundred and eighty-one patients (87%) were males and the median age was 34 years (range 19–72 years) and 69% of patients were intravenous drug users. The median CD4 cell count was 240/*μ*L (range 4–1100/*μ*L) and 57% of patients had a detectable HIV viral load.

MC was diagnosed in 53% of cases, followed by NS in 24% and LD in 14%. Advanced stages of disease were observed in 79% of patients and 76% had B symptoms. The overall extranodal involvement was 59% with bone marrow, spleen and liver involved in 38, 30, and 17, respectively. With the aim to evaluate the impact of HAART on clinical presentation and outcome of our patients, we split the series into two subgroups: in the first group we included those patients who received HAART since 6 months before the onset of HL (84 patients); in the second group we included those patients who never received HAART before the diagnosis of HL or less than 6 months (206 patients). Briefly, in comparison to never experienced HAART, patients in HAART before the onset of HL are older, have less B symptoms, a higher leukocyte, neutrophils count, and hemoglobin level. The following parameters were associated with a better overall survival (OS): MC subtype, the absence of extranodal involvement, the absence of B symptoms, and a prior use of HAART. Interestingly, three parameters were associated with a better time to treatment failure: a normal value of alkaline phosphatase, a prior exposure to HAART, and an international prognostic score less than 3 [38]. A similar study was carried out within the Spanish group GESIDA where the authors compared the clinical characteristics and outcome of 104 patients with HIV-HL, treated (83 patients) or not (21 patients) with HAART. No differences were found between the groups at baseline, but the complete remission (CR) rate was significantly higher in HAART group (91% versus 70%, *P* =  .023). The median overall survival was not reached in HAART group and was 39 months in no-HAART group (*P* =  .0089); the median disease-free survival (DFS) was not reached in HAART group and was 85 months in no-HAART group (*P* =  .129). Factors independently associated with CR were a CD4 cell count >100 cells/*μ*L and the use of HAART; CR was the only factor independently associated with OS [39].

The optimal therapy for HIV-HL has not been defined yet. Because most patients have advanced stages of disease, they have been treated with combination chemotherapy regimens but the CR rate remains lower than that of HL of the general population with the OS being approximately 1.5 years [8, 34–36]. Due to the low incidence of the disease, no randomized controlled trials have been conducted in this setting. However, several phase II studies have evaluated the feasibility and activity of different regimens. In a prospective trial, conducted within the GICAT between March 1989 and March 1992, 17 previously untreated patients with HIV-HL were treated with epirubicin, vinblastine, and bleomycin (EVB). Overall, CR was achieved in 53% of the total group, lasting a median of 20 months. The median OS for the group as a whole was 11 months and the 2-year DFS was 55% [40]. In an attempt to improve upon these results, from 1993 to 1997, a second prospective trial consisting of full dose EVB plus prednisone (EVBP regimen) and concomitant antiretroviral therapy (zidovudine or didanosine) was conducted. The results of this trial in which 35 patients were enrolled, showed a CR rate of 74% and a 3-year OS and DFS of 32% and 53%, respectively [41]. The AIDS Clinical Trials Group (ACTG) reported the results of a phase II study in 21 patients treated with ABVD chemotherapy for 4–6 cycles and primary use of G-CSF. Antiretroviral therapy was not used. The CR rate, on an intent to treat analysis, was 43% with an overall objective response rate of 62%. Median survival for all patients was 18 months [42]. Similar data have been reported in a small trial with only 8 patients enrolled [43]. The widespread use of HAART allows the use of more aggressive chemotherapeutic regimens. We used the Stanford V regimen, consisting of short-term chemotherapy (12 weeks) with adjuvant radiotherapy. From May 1997 to October 2001, 59 consecutive patients were treated in the framework of this prospective phase II study within the European Intergroup Study HL-HIV. Stanford V was well tolerated and 69% of the patients completed treatment with no dose reduction or delayed chemotherapy administration. The most important dose-limiting side effects were bone marrow toxicity and neurotoxicity. Eighty-one percent of the patients achieved CR and after a median followup of 17 months 33/59 (56%), patients are alive and disease-free. The estimated 5-year OS, DFS, and freedom from progression (FFP) were 59%, 68%, and 60%, respectively. Probability of FFP was significantly higher (*P* =  .002) among patients with an international prognostic score (IPS) of <2 than in those with IPS > 2, and the percentages of FFP at two years were 83% and 41%, respectively. Similarly, probability of OS was significantly different (*P* =  .0004), and the percentage of survival at three years was 76% and 33%, respectively for IPS < 2 and IPS > 2 [44]. Within the German group, the very intensive BEACOPP regimen has been tested in 12 untreated patients with a 100% of CR rate but a high incidence of opportunistic infections [45]. Recently, the results of a large prospective phase II study with ABVD have been published. Within a cooperative network in Spain, 62 patients with HIV-HL received the standard ABVD plus HAART. The scheduled six to eight ABVD cycles were completed in 82% of cases. Six patients died during induction, 54 (87%) achieved a CR, and two were resistant. The 5-year OS and event-free survival (EFS) probabilities were 76% and 71%, respectively. The immunological response to HAART had a positive impact on OS (*P* =  .002) and EFS (*P *=  .001) [46]. Interestingly, there are some anecdotic cases of use of HAART with antineoplastic intent in HIV-NHL, especially in primary effusion lymphoma. Recently a case of long-lasting response to HAART as the only therapy for HIV-HL has been reported suggesting the possibility to use this approach in selected cases [47]. Finally, within the GICAT we have recently concluded the accrual of 71 patients in a prospective phase II study aiming to evaluate feasibility and activity of a novel regimen including epirubicin, bleomycin, vinorelbine, cyclophosphamide, and prednisone (VEBEP regimen). Seventy percent of patients had advanced stages of disease and 45% had an IPS > 2. The CR was 67% and 2-year OS, DFS, TTF, and EFS were 69%, 86%, 59%, and 52%, respectively [48]. The results of the largest prospective studies are showed in Table 1.

 Because HIV-HL like other HL may progress or relapse, the use of high dose chemotherapy and autologous stem cell transplantation (ASCT) has been tested in this setting. Several data from different groups, including the GICAT, have demonstrated the feasibility of this approach that can be considered the gold standard in the salvage setting [49–53]. Different conditioning regimens, including or not total body irradiation, have been tested. Recently, the AIDS Malignancy Consortium demonstrated in a multinstitutional trial that a regimen of a dose-reduced high-dose chemotherapy, including cyclophosphamide and busulfan and ASCT, is well tolerated and is associated with favorable DFS and OS probabilities for selected patients with HIV-associated NHL and HL [54].

## 5. PET Scanning

Positron Emission Tomography using [18F]-Fluoro-2-Deoxy-D-Glucose (FDG-PET) was first introduced in the management of lymphomas in the early 1990s. It is now recognised as an important tool for staging and treatment response assessment in Hodgkin and non-Hodgkin lymphomas [55, 56]. Turning to predicting outcome, in the HIV-negative patients, residual FDG PET avidity after 2 cycles of ABVD has been shown to confer poor prognosis and therefore, has been proposed to guide future therapy [57, 58]. A negative PET scan after two cycles of ABVD predicted a 96% 2-year progression-free survival (PFS). Nearly 80% of the HL patients show a complete normalization of PET scan after two courses of ABVD [56]. This phenomenon, called “metabolic CR”, can be explained by the peculiar architecture and organization of the neoplastic tissue, where only few, scattered neoplastic cells (accounting for less than 1% of the total cellular population) are surrounded by a population of nonneoplastic mononuclear bystander cells. The latter cells are probably responsible for the immortalisation of Hodgkin and RS cells by stimulating cytokine production by other CD4+ lymphoid cells (paracrine loop) or by inducing cytokine production by the RS cells (autocrine loop). In cases presenting with bulky lesions at diagnosis, a negative early PET is often associated with a persisting bulky lesion of more or less unchanged size. The explanation might be that chemotherapy switches off the production of chemokines by the activated lymphoid cells, as described for TARC (Thymus and activation-related chemokine). The latter can be measured in the serum of HL patients and its level is correlated to the quality of treatment response: for patients in CR the levels are much lower than in patients with stable or progressing disease [59]. In contrast, PET scanning within the HIV framework can be problematic. Some preliminary reports suggested that FDG activity may correlate with detectable lymphoma [60, 61]. Although initial staging may not alter the treatment plan, it can provide additional information, assess possible involvement of critical location, and help foresee and possibly avoid further complications. However, the experience with PET scanning in the HIV-HL needs to be further studied. A baseline study is strongly recommended, since early PET interpretation is based on a site-to-site comparison of FDG uptake both before and after chemotherapy. Pitfalls are numerous and bring a particular challenge in these patients whom HIV-associated immunodeficiency predisposes to infection, as does the use of aggressive immunosuppressive chemotherapy regimens [62]. PET imaging requires cautious reading and pertinent clinical correlation to avoid diagnosing benign disease as malignant, such as, hypermetabolic foci seen in lung or oesophagus, which are common sites of HIV- and/or chemotherapy-promoted infections. Nodal FDG uptake can be observed in lymphoma, various infections (e.g., Mycobacterium avium intracellular, Mycobacterium tuberculosis, Herpes simplex virus, among others), and AIDS-related malignancies such as Kaposi sarcoma. In addition, stimulation of bone marrow following treatment with granulocyte colony stimulating factors induces a striking increase in FDG uptake in bone marrow. To take into account the possibility of minimal residual uptake, a semiquantitative approach has recently been proposed for interim PET interpretation in the context of an international protocol for advanced-stage HL [63, 64]. 

Finally, PET is useful for an accurate initial staging and it should be recommend to monitor treatment response, because PET appears to have a prognostic value, since a negative scan seems always associated with a favourable outcome. Significance of residual uptake at sites of disease needs further evaluation (e.g., biopsy). However, the use of FDG in the followup of HIV-HL patients who achieved CR cannot be routinely recommended and further studies are warranted prior to any definite conclusion.

## 6. Conclusions

The outcome of patients with HIV-HL has improved with better combined antineoplastic and antiretroviral approaches. The main important challenges for the next years are (a) to demonstrate in a randomized trial that ABVD is the standard regimen in HIV setting, (b) to validate the role of PET scan both in the staging and in the evaluation of response, (c) to better understand the interactions between chemotherapy and antiretroviral therapy, in order to reduce the toxicity of both approaches, (d) to evaluate the use of new drugs (i.e., bortezomib) in this setting, and (e) to evaluate the long-term toxicity of the treatment in cured patients.

## Figures and Tables

**Table 1:** Results of prospective studies in HIV-HL.

Regimen/Reference	no. of patients	Stages III-IV	Response rate	Complete remission rate	Overall survival
EBV [40]	17	88%	82%	53%	11 months
EBVP [41]	35	83%	91%	74%	16 months
ABVD [42]	21	81%	62%	43%	18 months
ABVD [43[	8	75%	100%	100%	43.5 months
Stanford V [44]	59	71%	89%	81%	59% at 5 years
BEACOPP [45]	12	92%	100%	100%	75% at 3 years
ABVD [46]	62	100%	87%	87%	76% at 5 years
VEBEP [48]	71	70%	78%	67%	69% at 2 years
